# Wide-scope biomedical named entity recognition and normalization with CRFs, fuzzy matching and character level modeling

**DOI:** 10.1093/database/bay096

**Published:** 2018-09-18

**Authors:** Suwisa Kaewphan, Kai Hakala, Niko Miekka, Tapio Salakoski, Filip Ginter

**Affiliations:** 1 Turku Centre for Computer Science, Turku, Finland; 2 Department of Future Technologies, University of Turku, Turku, Finland; 3 University of Turku Graduate School, Turku, Finland

## Abstract

We present a system for automatically identifying a multitude of biomedical entities from the literature. This work is based on our previous efforts in the BioCreative VI: Interactive Bio-ID Assignment shared task in which our system demonstrated state-of-the-art performance with the highest achieved results in named entity recognition. In this paper we describe the original conditional random field-based system used in the shared task as well as experiments conducted since, including better hyperparameter tuning and character level modeling, which led to further performance improvements. For normalizing the mentions into unique identifiers we use fuzzy character *n*-gram matching. The normalization approach has also been improved with a better abbreviation resolution method and stricter guideline compliance resulting in vastly improved results for various entity types. All tools and models used for both named entity recognition and normalization are publicly available under open license.

## Introduction

Named entity recognition and normalization are fundamental tasks in biomedical natural language processing (BioNLP) and finding solutions for them has been the main focus of various shared tasks organized within the BioNLP community (1, 2). BioCreative VI: Interactive Bio-ID Assignment (Bio-ID) track is one of the most recent shared efforts in developing these tools with the goal of automatically annotating text with the entity types and identifiers (IDs) for mentions such as genes and organisms, in order to facilitate the curation process. The task principally consists of two major subtasks: (i) named entity recognition (NER) and (ii) named entity normalization (NEN) (3).

On one hand, several machine learning-based approaches, such as support vector machines and neural networks, have been applied to NER tasks with varying entities ranging from genes to diseases, chemicals and anatomical parts (4, 5). The most recent successful approaches include conditional random field (CRF) classifiers and neural networks (5–7). The approaches for NEN, on the other hand, are largely based on string edit distance and term frequency-inverse document frequency (TF-IDF) weighted vector space representations with a variety of preprocessing approaches to remove the written variations (8, 9). Some neural approaches have also been suggested for the normalization task (10, 11) and, furthermore, strong results have been achieved by modeling NER and NEN tasks jointly (12).

The methods seen in the BioCreative shared task follow the same general trends; Sheng *et al*. (13) rely on a neural NER model with stacked recurrent layers and a CRF output layer. Their internal experiments showed promising results for this approach in comparison to traditional CRFs, but the model did not achieve state-of-the-art results in the official evaluation. However, their normalization system utilizing external Application programming interfaces (APIs) resulted in excellent performance. Another strong normalization approach is suggested by Dai *et al*. (14). Their system benefits from the full context of the target document instead of only relying on the caption text and attempts to find the most frequently mentioned identifiers in the case of ambiguous terms. They also suggest a convolutional neural model for the task but were not able to produce comparable results to their other approach. Moreover, Dai *et al*. only focus on organism entities, amajor disadvantage of their system.

**Figure 1:**
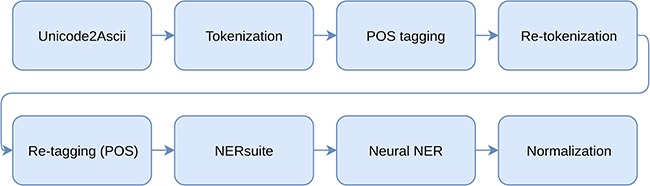
All processing steps included in the whole pipeline.

Our system, capable of recognizing six types of entities and assigning the corresponding identifiers, is based on CRF classifiers, fuzzy matching and a rule-based system (15). Our model achieved state-of-the-art results in the BioCreative shared task, being the best performing system in the NER subtask as well as the best performing system in normalization task for various entity types. Since the shared task we have improved the system in both of these subtasks even further. For the NER task we demonstrate an increased performance with an entity type-specific hyperparameter optimization protocol and character level modeling. For normalization, we improve the system by expanding the abbreviation resolution context to the full-text documents and by increasing the coverage of used ontologies. Both systems are publicly available at https://github.com/TurkuNLP/BioCreativeVI_BioID_assignment.

## Methods

Our system utilizes a pipeline of independent processing steps for solving the tasks of NER and NEN. The used processing modules as well as the used data are described in this section. The whole processing pipeline including preprocessing, named entity recognition and normalization is illustrated in Figure 1.

### Data

The Bio-ID data set consists of annotated figure panel captions extracted from 570 and 196 full-text documents as training and test data, respectively (3). The annotations include nine entity types; Protein/Gene (*Protein)*, Small molecules (*Molecule)*, Cellular compartment (*Cellular)*, Cell types and cell lines (*Cell)*, Tissues and Organs (*Tissue)*, Organisms and Species (*Organism),* microRNA (*miRNA),* BioAssay (*Assay*) and Protein Complexes (*Complex)*. The majority of annotated entities belong to the *Protein* class*,* contributing ∼55% of the whole training set, while *Complex* is the least-annotated entity type.

In these experiments we ignore entity types *Assay*, *miRNA* and *Protein Complex* as they were not evaluated in the BioCreative shared task. Hence, the total entity counts are 58 321, 7476, 6312, 11 213, 10 604 and 7888 for *Protein, Cellular, Tissue, Molecule, Cell and Organism*, respectively. We randomly partition the provided training data into a training and a development set, containing 455 and 115 documents, respectively. The development set is utilized in hyperparameter selection.

### Preprocessing

We preprocess the documents by using a publicly available tool for converting the character encodings to American Standard Code for Information Interchange (ASCII) (16). The characters with missing mapping, such as smiley faces and calendar symbols, are replaced with ‘-’ (dash). Subsequently we split the documents into sentences and further tokenize and part-of-speech (POS) tag them using GENIA sentence splitter (17), NERsuite tokenizer and NERsuite POS tagger modules (18), respectively.

Some of the documents contain incorrect word boundaries such as ‘mouseliverlysosomes’ which should have been written as ‘mouse liver lysosomes’. While the used tokenization tool is overall satisfactory, it is incapable of correctly splitting these spans into tokens. We thus resolve this by additional tokenization using the known tokens from the corresponding full-text document. Specifically, we split the tokens using the span of the longest-matching document tokens. To reduce the chance of mistakenly tokenizing correct tokens, we only re-tokenize the tokens that belong to noun phrases. Finally, we re-apply POS tagging to complete the data preprocessing.

### Ontologies and controlled vocabularies

We prepare a set of controlled vocabularies and ontologies to assist named entity recognition and normalization. List of concept names and ontologies we use includes ChEBI (19) and PubChem (20) (for *Molecule*), Entrez Gene (21) and Uniprot (22) (for *Protein)*, NCBI Taxonomy (23) (for *Organism)*, Uberon (24) (for *Tissue)*, Gene Ontology (25) (for *Cellular)* and Cellosaurus (http://web.expasy.org/cellosaurus/) and Cell Ontology (http://purl.obolibrary.org/obo/uberon.owl) (for *Cell*).

We preprocess the lists by removing non-alphanumeric characters and lowercasing the symbols. Specifically for NCBI Taxonomy, we additionally expand the ontology by adding the commonly used abbreviations for scientific names. For binomial nomenclature of names in species rank, we abbreviate the genus while the rest of the names such as species epithet, varieties, strains and substrains, remain the same. For example, ‘*Escherichia coli* O.1197’ is abbreviated as ‘*E. coli* O.1197’, ‘*E coli* O.1197’, ‘*Es. coli* O.1197’ and ‘*Es coli* O.1197’. This rule applies to all organisms, except for scientific names of organisms in Viruses and Viroids superkingdoms, since the scientific names do not usually follow binomial nomenclature but are in the form of *[Disease] virus* (26)*.* Acronyms are often used as abbreviated scientific names for viruses, for example ZYMV is the acronym of *Zucchini yellow mosaic virus*, and thus we also add acronyms to the ontology.

### NER

We train our NER system on the training set using NERsuite (http://nersuite.nlplab.org/)—a NER toolkit—and optimize model hyperparameters to maximize performance on our development set. The tokens are labeled with the IOB scheme, B denoting the beginning of an entity, I the following tokens of the same entity and O tokens not part of any entity. For our original shared task submission, we trained a single CRF model capable of detecting all possible entity types and used micro-averaged F1-score as the optimization metric, derived from the official evaluation script. To achieve higher performance in NER, we directly provide NERsuite with dictionaries through the built-in dictionary-tagging module with no further preprocessing or normalization. We compare the performance of different dictionaries on development data using default NERsuite hyperparameters. For the predictions on the test set, we merge the training and the development sets and re-train the CRF on this data using the best found hyperparameters.

Since the shared task we have conducted further experiments by training separate models for each entity type. Following the same approach as with the single model training scheme, each entity model is trained and optimized individually using development data for evaluating model performance. Although a single model can benefit from mutually supporting information between entity types, the regularization hyperparameter is global and selecting the other hyperparameters optimally for all entity types leads to a combinatorial explosion. Thus the advantage of separate models is that the hyperparameters can be selected independently for each entity type. The predictions on the test data are results of combining all the predictions from the six models without any further post-processing.

Although we try to handle subword entities by an heuristic re-tokenization, there are no guarantees that this leads to more suitable tokens for the given task. For example, the word ‘yeast’ can in certain contexts end up being re-tokenized as y e as t, with one to two characters in each token. To this end we also explore using a purely character-based model (27, 28), which does not rely on the correctness of the tokenization. For this experiment we train a neural convolutional bidirectional long short-term memory conditional random field (CNN-BiLSTM-CRF) model, which reads the input sentences a single character at a time and also predicts the IOB tags for each character separately. This model follows the general principle of Ma *et al.* (29) but does not rely on word embeddings. Each character in a sentence is represented with a latent feature vector, i.e. an embedding, and the convolutional kernels are applied on a window of five consecutive characters. As the convolutional kernels are applied only on the immediate context of the given character, a bidirectional long short-term memory (LSTM) layer is utilized for analyzing the larger context and longer dependencies between the characters. A CRF layer is used for the final outputs for better modeling of the dependencies between the output labels.

**Figure 2:**
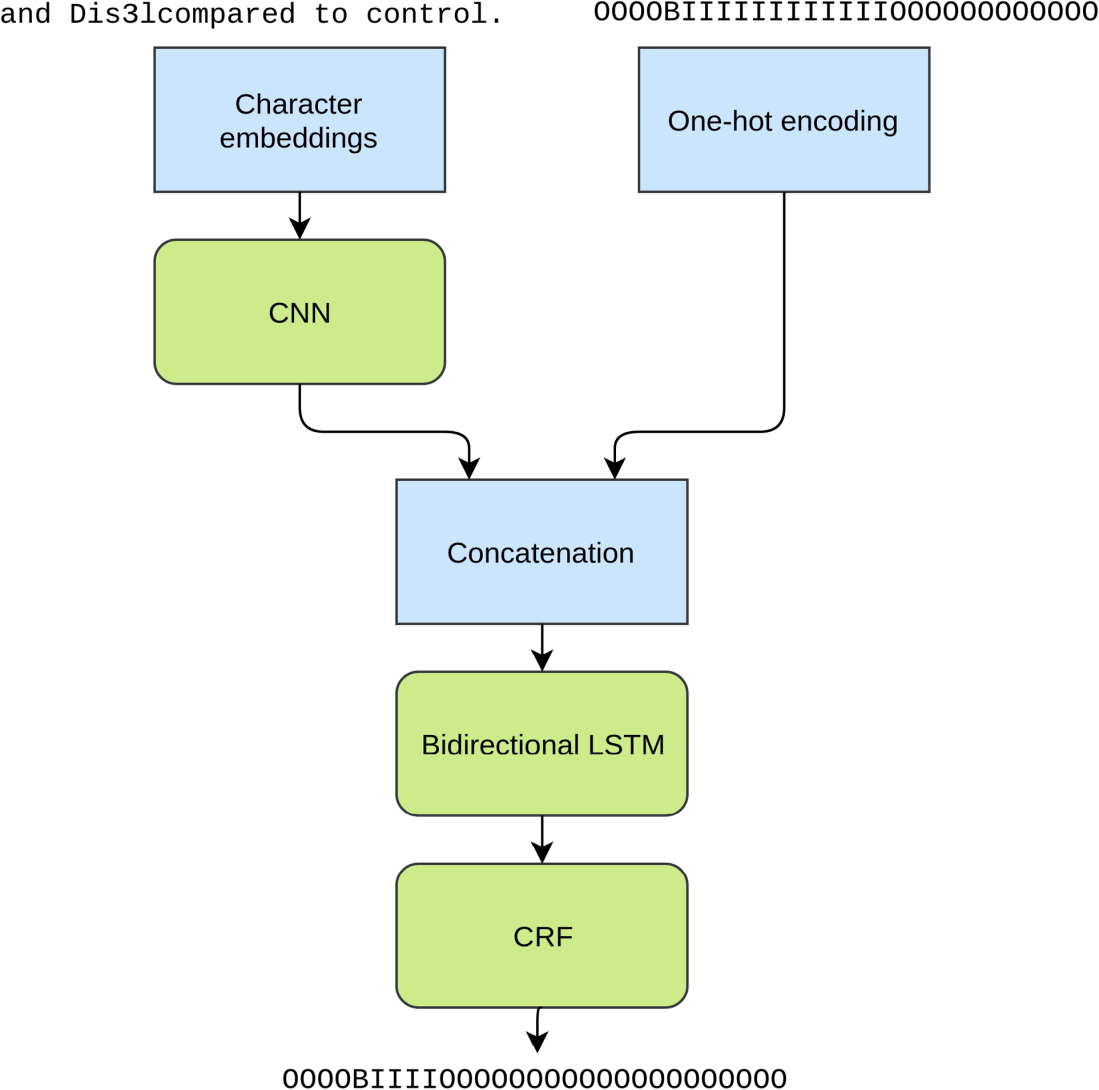
Illustration of the tested character level neural NER model. The inputs are a character sequence (a sentence) and the corresponding IOB tags from the NERsuite model converted to character level. The used example phrase demonstrates the tokenization issue as ‘Dis3lcompared’ is missing a word boundary and as a result is tagged as an entity as a whole in the NERsuite system. The neural model aims at detecting only the span ‘Dis3l’.

In addition to the character information we use the predictions from the original NERsuite model, converted to character level labels, as an additional input for the LSTM layer (Figure 2). Thus this approach can be seen as an ensemble method where the two models are stacked on top of each other. Providing this information is crucial as the CNN-BiLSTM-CRF model is not given any word level information and is unable to achieve good performance based solely on the characters. Thus the purpose of the neural model is not to learn the tagging task from scratch but to mainly correct the predictions made by the NERsuite model.

The benefit of such model is not only that it doesn’t depend on the tokens, but also that it is able to learn richer character level feature representations than NERsuite, which may improve generalizability on entity types such as *Proteins* which for a large part consist of short acronyms instead of the full names and tend to follow certain patterns.

As the neural model is trained on the same training data as NERsuite, but requires the NERsuite predictions as features, we simply apply the NERsuite model on its own training data. This inevitably leads to overly optimistic performance and the neural model learns to rely heavily on the NERsuite predictions. To mitigate this issue, the neural network inputs are regularized with the dropout method and the training is stopped once the performance is no longer improving on the development set. Due to the early stopping approach the final model is not trained with the merged training and development sets like NERsuite.

### NEN and disambiguation

Our normalization approach is primarily based on fuzzy string matching algorithm where both entity and ontology terms are converted into vectors using character *n*-gram frequencies. Cosine similarity is then used for calculating similarity between a detected entity and ontology terms. In this study, we use Simstring (30), a library for approximate string matching, to retrieve the ontology terms with highest cosine similarity with the queried entity. We utilize approximate string matching approach to all entity types except for *Protein*, which we instead map with exact string matching to the corresponding identifiers.

Prior to the similarity comparison the tagged entities resulting from the NER system are preprocessed by substituting the abbreviated names with full names using an abbreviation definition detector, Abbreviation Plus Pseudo-Precision (Ab3P) (31). In this study, we enhance our abbreviation detection to the provided unannotated full-text, rather than limiting to the annotated figure caption panels, as used in the previously submitted system. Subsequently, we preprocess the entities by the same approaches used on dictionaries and ontologies, i.e. lowercase all spans and remove punctuations, as described previously.

As some of the ontology terms cannot be uniquely linked to a single identifier, but correspond to multiple ones, our system thus selects an identifier randomly for *Cell, Cellular, Molecule* and *Tissue.* However, for *Molecule* and *Cell*, which can be mapped to multiple ontologies*,* we adopt the annotation guideline in selecting equivalent identifiers from different resources: for *Molecule,* ChEBI identifiers are preferred over PubChem, whereas Cellosaurus identifiers are primarily selected over Cell Ontology for *Cell.*

For most of the entity types, *Cell, Molecule, Cellular and Tissue,* each entity mention can be disambiguated and normalized independently. However, this approach is not applicable to *Organism* and *Protein* as the normalization of each mention depends on the normalization of previous mentions. For *Organism* and *Protein,* we hence develop two separate rule-based systems to uniquely assign an identifier.

For *Organism,* it is common to use a systematic abbreviation such as using genus name instead of binomial names to refer to the same species throughout the document. Subsequent mentions of those species, if abbreviated, should thus be normalized to an earlier mention of its corresponding binomial name. We use taxonomy tree and the following disambiguation rules to assign a taxon identifier to *Organism*. These rules are sequentially applied if the previous rule results in more than one identifier:

1. Take identifier with highest cosine similarity score and taxonomic rank under species, including subspecies, strain, variety and *no rank*.
2. Take identifier of a previously mentioned *Organism* if abbreviations match.
3. Take identifier of a previously mentioned *Organism* if acronyms match.
4. Take identifier of a previously mentioned *Organism* of the same genus.
5. Take identifier of a model organism of the same genus.
6. Take identifier of the most studied organism in PubMed Central Open Access section.
7. Take a random identifier.

*Protein* entities contain the most ambiguous names as the same protein names can be found in multiple organisms if they have the same function or shared sequence identity (32). Therefore, the information about the *Organism* is crucial for *Protein* entity normalization. We therefore employ the results of our *Organism* normalization system and use the taxon identifiers to disambiguate *Protein* entities. However, multiple taxon identifiers can be recognized in a single document, hence we adapt rule-based system proposed by (9) to generate candidate taxon identifiers for the *Protein.* The list of candidate taxon identifiers are ordered according to the following rules:

1. *Organism* mentioned inside *Protein* text span,
2. *Organism* mentioned before *Protein* within the same sentence,
3. *Organism* mentioned after *Protein* within the same sentence,
4. *Organism* mentioned in the previous caption and
5. *Organism* mentioned in the same document.

In addition, we perform query expansion to generate candidate *Protein* names to cover potential Uniprot and Entrez Gene symbol variations by using a stripping algorithm (33). The algorithm recursively removes common words*,* such as protein, gene, RNA and *Organism* names from *Protein* mentions to produce a *canonical form* which includes minimal symbols that are gene symbols in the Entrez Gene database. For instance, ‘p53 protein’ will result in ‘p53’. Finally, the canonical forms are subsequently lowercased and punctuation-removed. The list of candidate *Protein* names are then ordered by the string length.

For each taxon identifier, we use exact string matching to retrieve corresponding *Protein* identifier. The search starts with the longest candidate *Protein* name and stops when the identifier is found. In case of multiple identifiers, a random one is selected. For each taxon identifier, we use exact string matching to retrieve corresponding *Protein* identifier following the ordered list of candidate organisms. The search starts with the longest candidate *Protein* name and stops when the identifier is found. In case of multiple identifiers found in the current organism, a random one is selected. The search continues to the subsequent organism if no identifier is found for the current organism. For example, we first obtain the list of organisms for the mention of protein **ZEB1** from the sentence ‘Pearson correlation between **ZEB1** and MITF mRNA expression in 61 melanomacell lines available through the CCLE.’ As there are no mentions of other organisms within the sentence or previous caption, only human (NCBI Taxonomy:9606) and mouse (NCBI Taxonomy:10090) are found in the document. Starting from human, we then map ZEB1, the longest span of candidate gene names, to human gene identifier (NCBI Gene:6935) and the search stops as identifier is found for ZEB1.

## Result and discussion

The results presented in this section are based on the official evaluation scripts provided by the BioCreative shared task organizers. The NER task is evaluated on strict entity span matching, i.e. the character offsets have to be identical with the gold standard annotations. For the normalization task, only the normalized IDs returned by the systems are evaluated. The performance of the systems is reported as micro-averaged precision, recall and F-score oncorpus level.

### NER

Incorrect word boundaries can result in multiple types of entity annotations for a given token. For example, ‘mouseskinfibroblasts’ contains the annotations for *Organism, Tissue* and *Cell*. Since we train a single CRF-based model to recognize all types of entities, one token representing multiple entities causes the loss of training examples as NERsuite does not support multilabel classification. As mentioned in Method section, we resolve this issue by re-tokenizing the tokens using known tokens from the provided full-text document. The result for recovering the training examples is significant as tokenization from NERsuite alone yields roughly 97% of the annotations, while this step increases the number of annotations by additional 2 pp, equivalent to more than 2000 annotations. As a result, we recover more than 99% of the original annotations with *Organism* entity with the highest increase in coverage (Table 1).

**Table 1:** Comparison of annotation counts between tokenization approaches

***Re-tokenization***	***Protein***	***Cellular***	***Tissue***	***Molecule***	***Cell***	***Organism***
Without	97.178	99.772	95.951	96.107	97.099	93.691
With	99.187	99.866	99.842	99.424	99.559	98.921

The comparison of annotation counts between preprocessing with only NERsuite tokenization module (without) and with both NERsuite tokenization and additional tokenization (with). The numbers are percents of annotations compared to the provided data presented for each entity type.

While training a single model for all types of entities offers a relatively good performance, the model is tuned toward predicting *Protein,* the entity type with highest frequency in the training data. As a result, the performance of the model on other entities, such as *Cellular, Molecule and Tissue,* is lower than the overall performance. We thus resolve the issue by training NER model to detect entity types individually. The performance of these two training schemes for each entity type is shown in Table 2.

**Table 2:** Comparison of NER system on the development data

***Entity***	***Combined entity model***	***Independent entity model***	***CNN-BiLSTM-CRF model***
*Cell*	0.796 / 0.698 / 0.744	0.796 / 0.714 / 0.752	0.803 / 0.639 / 0.712
*Cellular*	0.759 / 0.611 / 0.677	0.710 / 0.682 / 0.696	0.725 / 0.633 / 0.676
*Protein*	0.771 / 0.726 / 0.748	0.755 / 0.738 / 0.746	0.833 / 0.779 / 0.805
*Organism*	0.878 / 0.696 / 0.776	0.872 / 0.757 / 0.810	0.856 / 0.713 / 0.778
*Molecule*	0.825 / 0.579 / 0.681	0.724 / 0.653 / 0.687	0.740 / 0.595 / 0.659
*Tissue*	0.816 / 0.566 / 0.668	0.750 / 0.696 / 0.722	0.730 / 0.607 / 0.663
***All***	**0.788 / 0.686 / 0.734**	**0.761 / 0.721 / 0.741**	**0.809 / 0.718 / 0.761**

Evaluation of the original model submitted to the shared task (*combined*), improved CRF model (*Independent*) and the neural character level model (*CNN-BiLSTM-CRF*) based on the official evaluation script with strict entity span matching on the development data. Numbers within cells are precision/recall/F-score.

As shown in Table 3, the independent entity models yield better F-scores for all entity types, except for *Protein*, by increasing recall while lowering precision*.* This is due to the fact that the best performing hyperparameters for each entity type are selected independently. In the single model approach the same hyperparameter values are used for all entity types, which results in them being dictated by the most common entity-type *Protein*. This can be seen during the optimization as hyperparameters for both independent model for tagging *Protein* and combined model are exactly the same. As a result, these parameters are thus suboptimal for tagging other entity types. While training a CRF-based model for multiple entity types can yield a better system performance, as the model can rely on dependencies between certain entity types, this is not the case in our experiment.

Even though optimizing the hyperparameters separately has the risk of overfitting on the development set, this does not seem to be the case in our experiments as training the independent models improves the performance of the system by 0.7 pp and 0.5 pp in F-score on development and test sets, respectively (Tables 3 and 4). The difference is most apparent on *Cell*, *Cellular* and *Tissue* entities with improvements of 1.5, 2.0 and 3.2 pp on the test set F-scores, respectively. Since these are less common entities than *Protein*, the influence on the overall score is not as pronounced.

**Table 3:** Official evaluation of NER system on the test data

***Entity***	***Combined entity model***	***Independent entity model***	***CNN-BiLSTM-CRF model***
*Cell*	0.783 / 0.708 / 0.743	0.767 / 0.749 / 0.758	0.769 / 0.641 / 0.699
*Cellular*	0.673 / 0.508 / 0.579	0.630 / 0.571 / 0.599	0.634 / 0.495 / 0.556
*Protein*	0.729 / 0.739 / 0.734	0.728 / 0.745 / 0.736	0.764 / 0.768 / 0.766
*Organism*	0.860 / 0.809 / 0.834	0.823 / 0.852 / 0.837	0.789 / 0.771 / 0.780
*Molecule*	0.775 / 0.587 / 0.668	0.661 / 0.681 / 0.671	0.667 / 0.595 / 0.629
*Tissue*	0.727 / 0.575 / 0.642	0.650 / 0.700 / 0.674	0.646 / 0.622 / 0.634
***All***	**0.747 / 0.694 / 0.720**	**0.719 / 0.730 / 0.725**	**0.739 / 0.702 / 0.720**

Evaluation of the original model submitted to the shared task (*combined*), improved CRF model (*independent*) and the neural character level model (*CNN-BiLSTM-CRF*) based on the official evaluation script with strict entity span matching on the test set. Numbers within cells are precision/recall/F-score.

**Table 4:** Official evaluation of NEN system on the development data

***Entity***	***Micro-averaged score***	
	***Our system (submitted to Bio-ID task)***	***Our system (this work)***
*Cell*	0.733 / 0.770 / 0.751	0.715 / 0.766 / 0.740
*Cellular*	0.478 / 0.493 / 0.485	0.462 / 0.491 / 0.476
*Protein*	0.445 / 0.315 / 0.369	0.410 / 0.360 / 0.383
*Organism*	0.724 / 0.669 / 0.695	0.802 / 0.668 / 0.729
*Molecule*	0.292 / 0.187 / 0.228	0.595 / 0.587 / 0.591
*Tissue*	0.598 / 0.672 / 0.633	0.592 / 0.680 / 0.633

Comparison of our normalization systems on development data with gold standard entities. Numbers within table cells are precision/recall/F-score.

With the neural approach a significant improvement of +2.0 pp over the underlying system can be seen on the development set. This improvement is solely caused by increased precision, which is intuitive as the purpose of the model is mostly to correct the existing predictions instead of detecting new ones. Unfortunately these promising results translate to a decrease of 0.5 pp on the test set compared against the NERsuite-based model. We have not done an exhaustive search over the neural network architectures or hyperparameters but mostly follow decisions made in previous studies. Thus we believe that the overfitting on the development data is caused by the early stopping procedure and could be alleviated by increasing the development set slightly at the expense of the training set.

### NEN and disambiguation

The performance of our normalization system is heavily dependent on the NER system performance since unrecognized and incorrect entity spans are automatically classified as false negatives and false positives, respectively. We thus evaluate our normalization system on the development set based on the gold standard entity mentions to compare the different approaches on different entity types.

As shown in Table 4, our normalization system submitted to the Bio-ID task performs moderately on *Cell, Cellular, Organism* and *Tissue,* where the F-score ranges from 0.485 to 0.751; however, the performance drops dramatically when evaluated on *Molecule* and *Protein.* In this study, we thus focus on improving the system for *Molecule* and *Protein* normalization*.*

For *Molecule,* we have improved our dictionary coverage and changed the rule to prefer assigning ChEBI identifier to the entity span as mentioned in the Method section. The latter change has the most significant impact on the system performance, increasing F-score by more than 25 pp.

For *Protein,* normalization is also slightly improved, however less significantly with only 1 pp increase in F-score. Unlike *Molecule,* our *Protein* normalization system primarily depends on the accuracy of both exact strings matching and the *Organism* normalization. As the former component remains unchanged, the improvement is solely determined by the latter, the *Organism* normalization. This influence can also be seen by using gold standard *Organism* mentions and identifiers: the precision, recall and F-score of *Protein* normalization increases to 0.451, 0.397 and 0.422, respectively. This overall 4 pp increase in F-score on *Protein* normalization demonstrates that correctly normalizing the *Organism* plays an important but only a limited role in our current *Protein* normalization system. Significant gains should be thus expected by improving the *Protein* normalization system itself.

For *Cell, Cellular* and *Tissue,* the performance of the system remains unchanged or slightly drops from our submission result. We suspect this is due to the lack of disambiguating rules if multiple matching identifiers are found. The result is thus probably an oscillation of accuracy for randomly selected identifiers.

We finally combine our normalization system with the newly developed NER systems and evaluate their combined performance on test data set. The performance of the current systems is compared against our previously submitted predictions and the results from the best performing systems.

As shown in Table 5, both of our systems developed in this work have relatively similar performance to our submitted system for all entity types, except for *Molecule, Cell* and *Organism.* For *Organism* and *Molecule,* the heightened performance can be attributed to positive effects of both NER and NEN systems. For *Cell*, however, the improvement on normalization score can be only explained by the improvement on NER system as our current normalization has introduced no further improvement, on the contrary actually lowering the F-score on the normalization of this entity type, while evaluated on gold standard entities. A mere increase of 1 pp in F-score on NER for *Cell* can subsequently translate into over 5 pp F-score improvement of the integrated system.

**Table 5:** Official evaluation of NER and NEN systems on the test data

***Entity***	***CRF-based combined entity model***	***CRF-based independent entity model***	***CNN-BiLSTM-CRF model***	***Best performing system***	***References***
*Cell*	0.600 / 0.576 / 0.588	0.630 / 0.664 / 0.647	0.674 / 0.610 / 0.641	0.784 / 0.557 / 0.651	Sheng *et al.* (14)
*Cellular*	0.456 / 0.371 / 0.410	0.404 / 0.423 / 0.413	0.391 / 0.376 / 0.383	0.550 / 0.450 / 0.495	Sheng *et al.* (14)
*Protein*	0.472 / 0.343 / 0.397	0.456 / 0.358 / 0.401	0.445 / 0.388 / 0.415	0.472 / 0.343 / 0.397	Our submitted system
*Organism*	0.668 / 0.667 / 0.667	0.753 / 0.725 / 0.739	0.761 / 0.703 / 0.731	0.660 / 0.883 / 0.756	Singh and Dai (34)
*Molecule*	0.244 / 0.240 / 0.242	0.439 / 0.489 / 0.462	0.460 / 0.456 / 0.458	0.587 / 0.473 / 0.524	Sheng *et al.* (14)
*Tissue*	0.531 / 0.490 / 0.510	0.427 / 0.565 / 0.486	0.451 / 0.542 / 0.493	0.531 / 0.490 / 0.510	Our submitted system

Comparison of our joint named entity recognition and normalization systems and the best performing systems in the shared task on the official test set. Numbers within table cells are micro-averaged precision/recall/F-score.

While the increase in NER system performance can be valuable for some entities, the effect of NER on normalization performance can be detrimental as well. As shown in Table 3, our NER systems with a slight increase in F-score on *Tissue* recognition has negative impact on normalization by lowering the F-score by 1–2 pp. One potential explanation is that the improved NER system also finds seemingly correct entities, which nevertheless are not considered correct according to the annotation guidelines. For example. in a phrase ‘smooth muscle basement membranes’, our system recognizes ‘smooth muscle’ as a *Tissue* entity and the normalization model is also able to find a corresponding identifier for it, but this is not considered to be an entity in the gold standard annotations as it is seen as a modifier for the ‘basement membranes’ *Cellular* entity. The combined NER model has plausibly learned this type of dependency between entity types and avoids many of these errors, whereas the independent entity type-specific NER models are not aware of the surrounding entities. Thus, it would be beneficial to take into account the normalization performance while optimizing the NER system as the recognized entities with missing or incorrect identifiers may be harmful for the real applications relying on the extracted information.

Our integrated system has moderate performance overall. Whereas the NER component achieves high F-scores compared to other systems submitted to the shared task, the normalization systems performance is still lagging for most entity types. While fuzzy string matching has good results on some of the entities, the result can be rather different as shown by the large variance in F-score (>33 pp) on different entity types. This signifies that the approach is not universally good for all types of entities, and other approaches, such as TF-IDF weighting, preprocessing and post-processing, steps should be also considered.

## Conclusions and future work

We approach BioCreative Bio-ID task by training a CRF-based model to recognize biomedical entities and we link them to their corresponding database identifiers using approximate pattern matching algorithm. For *Protein* and *Organism* entities, we utilize the ontology structure and surrounding context to disambiguate the entities with multiple identifier candidates.

Our CRF-based NER systems demonstrate a notable performance overall, achieving the best score out of all systems submitted to the Bio-ID task for all entity types, exceeding the performance of the second best systems by 8 to 18 percentage points depending on the entity type. In this extended study we have further improved the system with a more fine-grained hyperparameter optimization specific to each entity type. This approach leads to a significant improvement in performance for the less common entity types without sacrificing the overall performance.

We have also explored the possibility of correcting the predictions with a character level neural model stacked on top of the CRF model. The results suggest that such model can potentially offer considerable performance improvements, yet overfits easily to the development set. As a future work we will look into better ways of regularizing the network as well as consider the possibility of solely character level modeling, dismissing the ensemble approach.

Our NEN system submitted to the shared task demonstrated a lagging performance for *Protein, Cellular* and *Molecule* when compared with other entities. In particular, for *Cellular* entities the best performing systems are able to achieve up to 11 pp higher F-scores in the official evaluation. In this work, we have improved our system on all entity type by improving abbreviation resolution. For *Protein* normalization, even though the performance of the system is slightly increased by *Organism* assignment, we suspect that strict string matching criteria might be an important factor in limiting the system performance.

Our current normalization system is somewhat limited as it applies several manually generated rules which do not generalize to normalizing other entity types, hindering the ability of applying the same approach for entity types outside the scope of the BioCreative Bio-ID task. Thus developing a machine learning system that can be trained on the annotations of new entity type would be an ideal solution for the normalization task. Since the conventions of naming biomedical entities as well as the dependence on the surrounding context vary among entity types, a unified normalization system can be a challenging task.

## Notes

**Database URL:** https://github.com/TurkuNLP/BioCreativeVI_BioID_assignment
